# Enhancing methanol biotransformation for overproducing fatty acid derivatives

**DOI:** 10.1016/j.synbio.2022.10.003

**Published:** 2022-10-09

**Authors:** Xiangyin Chen, Jiaming Yu, Lixin Zhang

**Affiliations:** State Key Laboratory of Bioreactor Engineering, School of Biotechnology, East China University of Science and Technology, 130 Meilong Road, Shanghai, 200237, China

Excess amount of CO_2_ in the atmosphere is one of the major causes of the greenhouse effect and global warming [1]. CO_2_, a promising feedstock for bio-refinery, its efficient utilization is challenging due to its stable structure and low energy state [2,3]. However, small energetic compounds like methanol become a bridge and bond of efficient CO_2_ transformation [4,5]. Therefore, methanol biotransformation via microbial cell factories will pave the way for sustainable production of value-added chemicals.

Methanol metabolism is a complex and tightly regulated process, which generates toxic intermediate formaldehyde to hinder cell growth. Although artificial methanol metabolism could be achieved in model microbes such as *Escherichia coli* [[6], [7], [8]], the limited cell growth is far behind bio-productions. Alternatively, harnessing native methylotrophs could help methanol biotransformation toward high-level productions of high-value chemicals. Recently, Yongjin Zhou et al. reported the engineering of methylotrophic yeasts for efficient production of fatty acid derivatives from sole methanol [9,10].

First, *Ogataea polymorpha* is engineered to produce free fatty acids (FFA) by blocking β-oxidation via deleting fatty acyl-CoA synthase gene *FAA1* [9]. However, this *faa1*Δ strain failed to survive in methanol medium though it generated 983 mg/L of FFA from 20 g/L glucose. Rational metabolic rewiring to enhance the supply of cofactor and precursors, as well as methanol metabolism, failed in restoring the growth of *faa1*Δ strain from methanol. Then adaptive laboratory evolution (ALE) successfully obtained mutants with efficient cell growth and FFA production from sole methanol. Genome sequencing identified the mutation of two key genes, *LPL1* (putative lipase) and *IZH3* (membrane protein related to zinc metabolism), which were essential for restoring cell growth of *faa1*Δ strain in methanol and significantly increased the tolerance of wild-type strain against 50 g/L of methanol. Lipidomics revealed the deficiency in phospholipids hemostasis of *faa1*Δ strain, and the decreased levels of phosphatidylcholine (PC) and phosphatidyl-ethanolamine (PE) damaged peroxisomes. The leaked formaldehyde led to cell death via necrosis. Finally, the transcriptome-guided metabolic rewiring achieved a high level of FFA accumulation (up to 15.9 g/L) from sole methanol [9].

In the other study, Zhou et al. has reported engineering *Pichia pastoris* for high-level production of fatty acids and fatty alcohols from sole methanol [10]. Interestingly, disruption of fatty acyl-CoA synthase (*faa1*Δ, *faa2*Δ) did not result in cell growth deficiency, despite a slight growth inhibition with increased levels of formaldehyde and reactive oxygen species. This observation suggested that *P. pastoris* was more robust than *O. polymorpha* in the overproduction of FFA from methanol. Further enhancing the supply of precursor acetyl-CoA and cofactor NADPH (overexpressing gene *MmACL*, *ScIDP2*, *BbXFPK*, *CkPTA*), and fine-tuning methanol metabolism (overexpressing gene *DAS2*) enabled up to 23.4 g/L of FFA in fed-batch fermentations. Finally, a metabolic transforming strategy was developed to enable 2.0 g/L fatty alcohol from sole methanol, which avoids labor-intensive and time-consuming genetic manipulation [10].

The different behaviors between *O. polymorpha* and *P. pastoris* on bio-productions of FFA from sole methanol, suggest there was a big difference in methanol metabolism among various methylotrophic yeasts. Systematical mining of genome sequences may help to identify other key genes in regulating methanol metabolism, requiring to construct a better model to describe methanol toxicity during bio-productions (Fig. 1). The high-level production of fatty acid derivatives from sole methanol showed the strong potential of methanol biotransformation to value-added products for industrial applications.

**Fig. 1:**
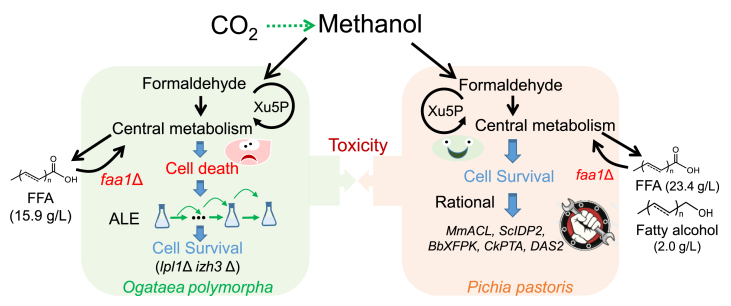
**Enhancing methanol biotransformation for efficient productions of fatty acid derivatives.** In *O. polymorpha*, overproduction of FFA strain resulted in cell death in a methanol medium. Adaptive laboratory evolution (ALE) and rational metabolic engineering enabled 15.9 g/L FFA from sole methanol. While FFA overproducing *P. pastoris* survived well in methanol and the global metabolic rewiring improved the production of FFA and fatty alcohols. Further systematically analyzing these two methylotrophic yeasts may help elucidate the molecule mechanism of methanol metabolism and toxicity.

## Declaration of competing interest

Nothing declared.
